# Equivalent Glycemic Load and Insulinemic Responses Elicited by Low-Carbohydrate Foods: A Randomized Trial in Healthy Adults

**DOI:** 10.1016/j.cdnut.2025.107594

**Published:** 2025-10-30

**Authors:** Thomas MS Wolever, Kevin Miller, Taylor Banh

**Affiliations:** 1 INQUIS Clinical Research, Inc., Toronto, ON, Canada; 2 Bell Institute of Health and Nutrition, General Mills, Inc., Minneapolis, MN, United States

**Keywords:** controlled trial, net-carbohydrate, Monte Carlo simulation, classification of dietary carbohydrates, glycemic index

## Abstract

**Background:**

The information on the Nutrition Facts Label may overestimate the available-carbohydrate (avCHO) content and glycemic impact of some low-carbohydrate foods containing novel carbohydrates.

**Objectives:**

The primary objective was to test the hypothesis that the glycemic impact of low-carbohydrate foods, quantified as equivalent-glycemic-load (EGL), measures their avCHO content accurately and precisely (within ±1g). The secondary objectives were to measure the glycemic and insulinemic responses elicited by 7 low-carbohydrate foods.

**Methods:**

Healthy overnight-fasted adults (*n =* 25) consumed 20.8 g or 5.2 g avCHO from white bread (WB20.8, WB5.2, respectively), or 1 of 7 test-products in random order; each subject tested WB20.8 twice. Plasma-glucose responses were measured in all subjects with serum-insulin measured in 10. For each subject, 20.8 × *T*/*W* was calculated [*T* = incremental area under the glucose curve (iAUC) after the test-food, *W* = mean iAUC after WB20.8]; the mean of the resulting values (excluding outliers) was the test-food EGL.

**Results:**

The expected EGL of WB5.2 was 5.2 g and the measured value was 4.0 g (95% margin of error = 0.6 g). On the basis of the food-label, the test-products contained 3–12 g avCHO (total-carbohydrate minus dietary-fiber). However, because 5 of the test-products contained allulose, which is not included in dietary-fiber and not quantified on the food-label, their content of netCHO (avCHO minus allulose) ranged from 3 to 6 g; even so, their EGL values varied from just 0.6 to 2.4 g. The mean insulin responses elicited by the test-products were positively related to their protein content, but none differed significantly from that elicited by WB5.2.

**Conclusions:**

The results support the hypothesis that the EGL measure is accurate and precise to within ∼±1 g. The EGLs of the 7 test-products were 20%–90% less than expected from their food-labels. The test-products elicited small insulin responses that were positively related to their protein content.

This trial was registered at clinicaltrials.gov as NCT05870891 (https://clinicaltrials.gov/study/NCT05870891).

## Introduction

The global low-carb market size, currently valued at USD 14.5 billion, is projected to reach a value of USD 34.6 billion by 2032 [1]. Low-carb products contain low amounts of carbohydrate and increased amounts of protein and healthy fats. People may adopt low-carbohydrate diets to assist with weight management and/or to reduce blood lipids and spikes of postprandial blood glucose [1]. To assess the glycemic impact of low-carb foods, health professionals and consumers may use the food’s macronutrient content shown on the Nutrition Facts Label (NFL).

In North America, the NFL lists values for total-carbohydrates (termed “carbohydrate”) and dietary fiber (termed “fiber”). “Carbohydrate” includes both available carbohydrates (avCHO) and unavailable carbohydrates (“fiber”). AvCHOs are digested and absorbed in the small intestine and metabolized in the body as glucose [2], whereas unavailable carbohydrates (“fiber”) are not. Thus, only avCHO raises blood glucose [2]. The glycemic response elicited by conventional carbohydrate foods (e.g. sugars, fruit, grains, tubers, legumes) is largely determined by the source and amount of avCHO they contain [[3], [4], [5]]. In such foods, the difference between the NFL values for “carbohydrate” and “fiber” is a reasonable estimate of avCHO. However, this calculation may overestimate the amount of avCHO in low-carb foods in which avCHOs have been replaced by unavailable novel carbohydrates that are not classified as “fiber.” Such carbohydrates may be used as food ingredients because they have a sweet taste that consumers desire without the use of conventional sugars or “artificial” sweeteners.

“Fiber” on the NFL includes some unavailable carbohydrates (e.g. polydextrose and inulin), but not all, such as maltitol, which is ∼60% unavailable [6], and erythritol and allulose, which are almost totally unavailable because, although well absorbed, they are not metabolized and excreted in the urine [7,8]. In foods containing such ingredients, the difference between the NFL values for “fiber” and “carbohydrate” may overestimate the avCHO content, and potential glycemic impact. Thus, we developed a method to quantify the glycemic impact of low-carb foods termed equivalent glycemic load (EGL) [9]. EGL is the grams of avCHO from white bread with the same glycemic impact as 1 serving of food. The original EGL method was accurate to within 1 g avCHO [9] and involved each subject consuming 0, 5, 10, and 20 g avCHO portions of white bread to create a dose-response curve. However, because the dose-response to avCHO is virtually linear between 0 and 20 g [9], we simplified the EGL method by having subjects test a 20-g avCHO portion of white bread twice.

Low-carb foods may contain large amounts of protein and/or fat. Protein or fat, when consumed alone, elicit a small insulin response [10,11]. When added to avCHO, they reduce the glycemic response in a dose-dependent fashion [12,13] by delaying gastric emptying [14] and increasing postprandial insulin [13]. Studies assessing the insulinemic impact of low-carbohydrate foods [15,16] do not show how varying the amounts of protein and fat in low-carb foods affect postprandial glucose and insulin responses.

The primary objective of this study was to test the performance of the modified EGL method. We hypothesized that the measured EGL value of a portion of white bread containing ∼5 g avCHO would be accurate and precise to within ±1 g. The secondary objectives were to measure the EGL of 7 low-carb foods and to obtain preliminary information about whether variations in their protein and fat contents were associated with their postprandial glucose and insulin responses.

## Methods

The study had an open-label, randomized block, crossover design and was carried out at INQUIS Clinical Research, Inc. (INQUIS). It was first posted on clinicaltrials.gov as NCT05870891 on 13 May 2023. The study description was corrected on 30 May 2023. The first subject was screened on 15 June 2023. The last subject visit was on 3 October 2023.

We aimed to recruit enough healthy adults from the pool of previous participants in studies at INQUIS who had given permission to be contacted for future studies or from online advertising to obtain 25 who successfully completed the study. Blood for measurement of both glucose and insulin was collected from the first 10 subjects who had done so before, whom we knew were able to collect reliably the required amount of blood quickly (within 1–2 min of the required time) and were willing to do so; blood for measurement of glucose only was collected from the other 15 subjects. To be included, participants had to be generally healthy adults aged 18–75 y, inclusive, who were not pregnant or lactating. and had no history of diabetes. They had to be willing to abstain from unusual strenuous exercise and consuming alcohol for 24 h before study days and to refrain from smoking tobacco or marijuana for 12 h before and during study visits. There was no restriction on body weight. Details of the inclusion/exclusion and withdrawal criteria are given in Supplemental Material, Section 1.1.

### Ethics and consent

The final protocol and consent form was approved on 12 May 2023 by the Advarra IRB (protocol number Pro00071556). Participants were not enrolled until they had had the study procedures explained to them, had a chance to ask questions, and had given their voluntary consent by signing the consent form. Consent was obtained by inviting potential participants to come to INQUIS to have the study procedures explained to them and be given a copy of the consent form to review. They could either sign the consent form then, take it away to sign later, or decline to participate. Participants were encouraged to ask any questions they had and not to sign the consent form until all their questions had been answered to their satisfaction.

### Procedures

Participants who had provided informed consent attended a screening visit to determine eligibility; they were asked questions to assess medical history, medication use, and to have their height and weight measured. The tools used to determine sex, gender identity, and ethnicity are shown in Supplemental Material, Sections 1.2 and 1.3.

Eligible participants were studied on 10 separate days over a period of 4–12 wk with a washout of ≥1 d between visits. Participants were asked to refrain from drinking alcohol, to avoid unusual levels of food intake and physical activity the day before each study visit, and to avoid smoking tobacco or marijuana for 12 h before each study visit. If participants had any signs or symptoms of an active infection within 5 days before the test visit, the visit was rescheduled until all signs and symptoms had resolved and any treatment (e.g. antibiotic therapy) had been completed for ≥5 d before the test visit. If the participant had not complied with the preceding experimental conditions, the test was not carried out and was rescheduled for another day.

On each test day, participants came to INQUIS in the morning after a 10–14-h fast consuming nothing other than water. After reviewing drug and medical history and confirming that the participant had complied with the instructions regarding alcohol, food intake, physical activity, and smoking the day before, participants were weighed, and 2 fasting finger-stick blood samples at 5-min intervals were collected. Then participants started to consume a test-meal and were asked to consume the entire test-meal and beverage (see below) at an even pace over a period of ∼12 min. A timer was started at the first bite/sip and further finger-stick blood samples were obtained at 15, 30, 45, 60, 90, and 120 min. Subjects remained seated quietly during the 2 h of the test. After the last blood sample had been obtained, subjects were offered a snack and could leave.

### Test-meals

Test-meals consisted of 1 serving of each of the 7 test-products, a portion of white bread containing 5.2 g avCHO (WB5.2) or a portion of white bread containing 20.8 g avCHO (WB20.8). The test-products were sriracha crisp (SirCr), cheddar crisp (ChdCr), almond blueberry butter bar (ABBB), peanut butter dark chocolate bar (PBDCB), almond dark chocolate bar (ADCB), peanut butter cereal, and cinnamon almond cereal (CinnCer). The test-products were produced by General Mills but are not commercially available. The macronutrient composition of WB5.2, WB20.8 and the test-products are shown in Table 1. The test-meals were divided into 2 blocks; block 1 consisted of 3 bars, WB5.2 and WB20.8 in random order; block 2 consisted of 2 cereals, 2 crisps, and WB20.8 in random order. Randomization was performed using the RAND() function of Excel (Microsoft Corp) by TMSW and sequences were assigned to subjects in order of their attendance for the first study visit. This was an open-label study with no concealment.

**TABLE 1:** Composition of the test-meals consumed by the participants

Test-meal	Abbreviation	Weight (g)	Energy (kcal)	Fat^1^ (g)	Protein (g)	Carbohydrate (g)			
						Total	Fiber	Avail^2^	Allulose^3^
White bread (20.8 g avCHO)^4^	WB20.8	∼44	106 ± 4	0.4 ± 0.1	3.7 ± 0.5	22.0 ± 0.6	1.1 ± 0.1	20.8 ± 0.6	—
White bread (5.2 g avCHO)^4^	WB5.2	∼11	27 ± 1	0.1 ± 0.0	0.9 ± 0.1	5.5 ± 0.2	0.3 ± 0.0	5.2 ± 0.1	—
Sriracha crisp^5^	SirCr	28	140	10 (1.0)	10	5	2	3	0
Cheddar crisp^5^	ChdCr	28	140	10 (1.5)	10	5	2	3	0
Almond blueberry butter bar^5^	ABBB	40	210	16 (3.5)	8	12	4	8	3
Peanut butter dark chocolate bar^5^	PBDCB	40	210	17 (4.0)	8	11	5	6	4
Almond dark chocolate bar^5^	ADCB	40	210	17 (3.5)	8	11	4	7	3
Peanut butter cereal^5^^,^^6^	PBCer	61	247	11 (1.5)	34	14	2	12	6
Cinnamon almond cereal^5^^,^^6^	CinnCer	61	247	11 (0.8)	34	14	2	12	8

Data for test-products provided by the sponsor.

1 Values in brackets indicate the amount of saturated fat.

2 Avail = available carbohydrate (total carbohydrate minus dietary fiber).

3 Allulose is a noncaloric (∼0.4 kcal/g) and nonglycemic carbohydrate. The amount of glycemic carbohydrate (i.e. net-carbohydrate) in each food was calculated as available carbohydrate minus allulose.

4 Weights of WB5.2 and WB20.8 are approximate because loaf weights varied by ∼±2%. Data for WB5.2 and WB20.8 were based on proximate analysis (means ± SD of 4 determinations in 2021–2023).

5 The amounts shown are the weight and composition of 1 serving of each food.

6 Milk was not added to cereals.

Each subject chose a beverage (water, coffee, or tea with or without 30 mL of 2% milk and a noncaloric sweetener if desired) to consume with each test-meal; the beverage chosen was kept the same for all test-meals.

White bread was baked in a bread maker in loaves containing 500 g avCHO. The ingredients for each loaf (510 mL warm water, 694 g all-purpose flour, 14 g sugar, 8 g salt, and 13 g yeast) were placed into the bread maker according to instructions, and the machine turned on. After the loaf had been made, it was allowed to cool to room temperature and weighed. After discarding the crust ends, the remainder was divided into portions containing 5 g avCHO or 20 g avCHO (based on proximate analysis of previous loaves baked using the same recipe), frozen before use and reheated in a microwave before consumption. On the basis of the mean results of proximate analysis of 2 samples of bread baked for this study combined with 2 previous results, the bread portions used contained 5.2 g and 20.8 g avCHO (Table 1).

### Blood sample collection and analysis

Blood was obtained by finger-stick; each blood sample consisted of 5–6 drops collected into heparin:fluoride microvettes (Sarstedt AG) for plasma-glucose analysis and 5–6 drops collected into empty microvette tubes for serum-insulin analysis. Microvettes containing blood for glucose were mixed by inverting the tube several times, centrifuged for 5 min, and stored at 4°C before plasma-glucose analysis within 5 d using the Vitros 350 Chemistry System (Vitros 350 Chemistry System, Ortho Clinical Diagnostics). Microvettes containing blood for insulin were left at room temperature to allow the blood to clot then centrifuged and the serum transferred to labeled polypropylene tubes and stored at −70°C before analysis of insulin using the Human Insulin EIA Kit (catalog # 80-INSHU-E10.1, Alpco Diagnostics). Glucose and insulin concentrations were transferred from the analyzers to an Excel spreadsheet electronically.

### Randomization

Randomization was based on a balanced, Latin-square design. This study had 2 blocks of 5 treatments; block #1 consisted of the 3 bars, WB20.8 and WB5.2 and block #2 consisted of the 2 cereals, the 2 crisps, and WB20.8. Subjects completed block #1 first and then went on to block #2; a comparison of the results for the 2 WB20.8 tests provides an assessment of any significant order effect (or lack thereof). Within each block of 5 treatments, 2 blocks of 5 sequences are required for a balanced design; this provides randomization for 10 subjects. However, with the 25 subjects in this study, 1 block of 5 is required along with 2 blocks of 10, so the orders were not perfectly balanced. The sequences in each of the 2 blocks of 10 sequences and the 1 block of 5 sequences were randomly ordered separately using the RAND() function in Excel by TMSW. The process was repeated for the second block of 5 treatments. Because this was an open-label study, the sequences were printed in tabular form and assigned to subjects by the research assistants as they arrived for their first visit; the subject ID was written on the chart along with the date each treatment was completed.

### Calculations

Incremental areas under the curves (iAUC) for glucose and insulin were calculated using the trapezoid rule, ignoring the area beneath the baseline. For the iAUC calculation, fasting glucose and insulin were taken to be the means of their first measurements at times -5- and 0-min. The SDs of the difference between duplicate measures of glucose in the 0-min sample (analytical variation abbreviated SDa), the first measures of glucose or insulin at -5- and 0-min (a combination of minute-to-minute and analytical variation), and glucose iAUC after WB20.8#1 and WB20.8#2 were calculated as follows:

$$\text{SDa}=\sqrt{(}\sum d^{2}/2n)$$

where *d* is the difference for each pair of measures and *n* is the number of pairs. The EGL of each test-food and WB5.2 was calculated for each subject as follows:

EGL=20.8×T/W

where *T* was the iAUC elicited by the test-food or WB5.2, *W* was the mean iAUC after WB20.8, respectively, and 20.8 the amount of avCHO in WB20.8. The mean and SD of the EGL values of all subjects who completed the trial were calculated and values >1.96 × SD from the mean were excluded as outliers. Outliers were excluded because glycemic responses within-individuals vary randomly from day-to-day and are normally distributed [17]. The EGL calculation is a constant times the ratio of 2 independently variable glycemic responses. The distribution of such ratios is skewed to the right [17,18,19], an effect that artificially increases the mean and SD. Outliers were excluded to mitigate this effect. The mean of the individual EGL values after excluding outliers was the EGL of the product; 95% confidence intervals (CIs) were calculated using the t-distribution.WB5.2 was included as a test-meal to assess the performance of the EGL method; because WB5.2 contained 5.2 g avCHO from white bread, by definition, its EGL equals 5.2 g. We hypothesized that the EGL method is accurate and precise to within ∼±1 g.

### Monte Carlo EGL simulation

We performed a Monte Carlo simulation to determine the effect of the observed magnitude of within-individual variation of glycemic responses on the distribution of EGL values and the number and distribution of excluded outliers. To remove between-subject variation from the simulation, the glucose iAUCs elicited by WB20.8 and WB5.2 were fixed at values of 100 and 25, respectively. The magnitude of within-individual variation in the simulation was accounted for by assigning WB20.8 an SD of 20.2 [the observed coefficient of variation (CV) of within-individual variation of the iAUCs for the 2 tests of WB20.8, 28.6%, divided by √2 (28.6/√2 = 20.2) to account for the fact that the mean iAUC of the 2 tests of WB20.8 in each subject was used to calculate EGL]. WB5.2 was assigned a mean iAUC of 25 because the dose-response curve for glucose iAUC on dose of avCHO is virtually linear between 0 and 20 g avCHO [9], and because the avCHO content of WB5.2 was 25% of that of WB20.8, its iAUC was taken to be 0.25 × 100 = 25. WB5.2 was assigned an SD of 15 based on the results of a previous study [9] in which the CV of within-individual variation of glycemic responses elicited by 5 g avCHO from white bread, 65%, was 2.1 times that for 20 g avCHO from white bread, 31%. Thus, the within-individual CV for WB5.2 was taken to be 28.6% × 2.1 = 60% of its iAUC (0.6 × 25 = 15). Using the Excel formula NORMINV(RAND(),X,Y), where X and Y for WB20.8 were 100 and 20.8, and X and Y for WB5.2 were 25 and 15, respectively, normally distributed random numbers were generated to represent the glycemic responses elicited by WB20.8 and WB5.2 in 3000 subjects. Each subject’s EGL was calculated as 20.8 × B5/B20 where B5 and B20 were the simulated iAUCs for WB5.2 and WB20.8, respectively and 20.8 is the amount of avCHO in WB20.8. Simulated iAUC values <0 were replaced by 0, because iAUC cannot be <0. The 3000 simulated values for WB20.8 and WB5.2 were used to calculate 3000 simulated EGL values that were divided sequentially into 120 groups of 25. The mean and SD of the simulated EGL for each group of 25 were calculated both before and after removing outliers (values >1.96 × SD from the mean). The distributions of the 3000 simulated iAUC values for WB20.8 and WB5.2, the 3000 individual simulated EGL values, and the 120 means for the simulated EGL values were determined. The number of simulated EGL outliers excluded was compared with the number of observed EGL outliers excluded for WB5.2 and the 7 test foods.

### Power and statistical analysis

Because there are no published studies using the simplified method for determining EGL we used, there is no information about the number of subjects required to test the primary endpoint; 25 was chosen as being able to provide 50%–70% more power than the 10–12 subjects typically used for measuring EGL or glycemic index (GI) [9,19].

Glucose iAUC values for WB20.8#1, WB20.8#2, WB5.2, and the 7 test-products in all 25 subjects who completed the trial were subjected to repeated measures analysis of variance using the general linear model (ANOVA) examining for the main effect of test-meal. If a significant effect was present, the individual means were compared using Tukey’s test with the criterion for significance being 2-tailed *P* < 0.05. Separate ANOVAs were performed to compare the glucose and insulin iAUC values in the 10 subjects in whom both glucose and insulin were measured.

To assess the effect of varying protein and fat contents on glucose and insulin responses among the 7 test-products, the relationships between various measures of food composition and glucose and insulin responses were assessed by regression analysis.

## Results

Twenty-six subjects were recruited but 1 dropped out after 3 visits because of a change in work schedule, leaving 25 who completed the study (study diagram in Supplemental Figure 1). The age, sex, ethnicity, height, weight, BMI, and medication use among the 10 participants in whom both glucose and insulin were measured did not differ significantly from those among the 15 participants in whom only glucose was measured (Table 2). Details of medications used by participants, the 6 adverse events in 5 participants (none were serious, and none were related to the study product), the 21 minor protocol deviations in 7 participants (none felt to have an important effect on the results) and the performance and completeness of the glucose and insulin analyses are shown in Supplemental Material, Sections 2.1–2.5.

**TABLE 2:** Summary of demographic characteristics of participants

Group	Sex^1^ (M:F)	Ethnicity				Age^2^ (y)	Height^2^ (cm)	Weight^2^ (kg)	BMI^2^ (kg/m^2^)
Glucose and insulin	7:3	WN-2	SA-4	SEA-2	EA-1	33.1 ± 13.2	169 ± 8	75.3 ± 12.9	26.2 ± 4.1
		LA-1	BC-0	WE-0	M-0	(20–62)	(160–198)	(55.9–89.8)	(19.9–33.7)
Glucose only	6:9	WN-3	SA-0	SEA-2	EA-3	39.0 ± 15.6	170 ± 9	77.9 ± 16.3	26.9 ± 4.8
		LA-2	BC-2	WE-2	M-1	(21–64)	(159–189)	(56.5–106)	(19.6–37.5)
*P*^3^	0.14	0.19				0.34	0.94	0.69	0.69
Combined	13:12	WN-5	SA-4	SEA-4	EA-4	36.6 ± 14.7	170 ± 9	76.8 ± 14.8	26.6 ± 4.5
		LA-3	BC-2	WE-2	M-1	(20–64)	(159–198)	(55.9–106)	(19.6–37.5)

Abbreviations: BC, Black Caribbean; EA, East Asian; LA, Latin American; M, mixed Middle Eastern/White European; SA, South Asian; SEA, Southeast Asian; WE, White European; WN, White North American.

1 Sex at birth and gender identity were the same for all subjects.

2 Values are means ± SD (range).

3 Significance of the difference between 10 glucose/insulin participants and 15 glucose only participants by chi-square test or unpaired t-test.

### Plasma-glucose responses in all 25 participants

**FIGURE 1:**
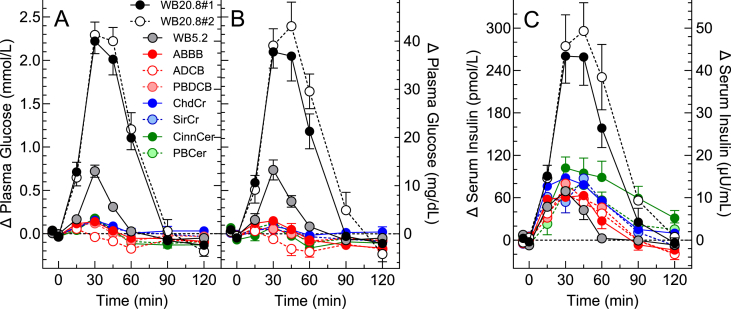
Plasma glucose and serum-insulin increments. Values are means ± SEM (error bars not shown if they overlap or are smaller than the symbol). (A) Plasma glucose concentrations in 25 subjects. (B) Plasma glucose concentrations in 10 subjects. (C) Serum-insulin concentrations in the same 10 subjects as shown in (B). ABBB, almond blueberry butter bar; ADCB, almond dark chocolate bar; ChdCr, cheddar crisp; CinnCer, cinnamon cereal; PBCer, peanut butter cereal; PBDCB, peanut butter dark chocolate bar; SirCr, sriracha crisp; WB20.8#1 and WB#20.8, 20.8 g avCHO portions of white bread tested in the first and second phases, respectively; WB5.2, 5.2 g avCHO portion of white bread.

The mean fasting glucose for the 10 treatments varied by <0.1 mmol/L (minimum (mean ± SEM) 5.41 ± 0.07; maximum, 5.49 ± 0.07mmol/L, *P* = 0.94). The incremental glycemic responses elicited by the 10 test-meals are shown in Figure 1A (plasma-glucose concentrations are shown in Supplemental Figure 2A). The mean iAUC after WB20.8#1 was similar to that after WB20.8#2, and both were significantly greater than those after all the other test-meals (Table 3). The mean ± SEM iAUC for WB20.8#1 and WB20.8#2 was 105.5 ± 6.2 mmol × min/L; the SDa of the duplicate measures was 30.2 mmol × min/L for an analytical CV of 28.6%. Mean iAUC after WB5.2 was significantly greater than those after all 7 test-products (Table 3). Among the 7 test-products, mean glucose iAUC after ChdCr was significantly greater than those after CinnCer and ADCB and mean glucose iAUC after SirCr was significantly greater than that after ADCB (Table 3).

**TABLE 3:** Incremental areas under the glucose curve (iAUC) and equivalent glycemic load

Test-meal	Abbreviation	Glucose iAUC (mmol × min/L)	Equivalent glycemic load (g)	
			All values	Outliers excluded∗
White bread #1 (20.8 g avCHO)	WB20.8#1	101.1 ± 7.0^a^	—	—
White bread #2 (20.8 g avCHO)	WB20.8#2	110.0 ± 8.0^a^	—	—
White bread (5.2 g avCHO)	WB5.2	21.6 ± 2.4^b^	4.3 ± 0.4 [3.4, 5.2]	4.0 ± 0.3 [3.3, 4.6]^1^
Sriracha crisp	SirCr	11.1 ± 2.4^cd^	2.1 ± 0.5 [1.2, 3.1]	1.9 ± 0.4 [1.1, 2.6]^1^
Cheddar crisp	ChdCr	13.7 ± 2.5^c^	2.6 ± 0.5 [1.7, 3.6]	2.4 ± 0.4 [1.5, 3.3]^1^
Almond blueberry butter bar	ABBB	9.4 ± 1.7^cde^	1.8 ± 0.3 [1.2, 2.4]^c^	1.5 ± 0.3 [1.0, 2.1]^2^
Peanut butter dark chocolate bar	PBDCB	9.0 ± 1.6^cde^	1.7 ± 0.3 [1.1, 2.4]	1.4 ± 0.3 [0.9, 2.0]^2^
Almond dark chocolate bar	ADCB	4.7 ± 1.3^e^	0.8 ± 0.2 [0.4, 1.3]	0.6 ± 0.2 [0.3, 1.0]^2^
Peanut butter cereal	PBCer	9.7 ± 2.5^cde^	1.9 ± 0.5 [0.9, 2.9]	1.6 ± 0.4 [0.8, 2.3]^1^
Cinnamon almond cereal	CinnCer	7.8 ± 1.6^de^	1.5 ± 0.3 [0.9, 2.2]	1.3 ± 0.3 [0.8, 1.9]^1^

Results are mean ± SEM [95% confidence interval] in 25 subjects.

^abcde^Means not sharing the same letter superscript differ significantly by 2-tailed Tukey’s test (*P* < 0.05).

^1,2^The number in the superscript indicates the number of outliers excluded.

∗ As indicated in the protocol, the final EGL values are those after excluding outliers.

### EGL

The mean ± SEM {SD} (95% CI) measured EGL of WB5.2 in all 25 subjects was 4.3 ± 0.4 {2.2} (3.4, 5.2) g; after excluding 1 outlier 3.5 × SD from the mean, the EGL was 4.0 ± 0.3 {1.5} (3.3, 4.6) g (Table 3).

After excluding 1 or 2 outliers for each test-product, their mean EGL values ranged from 0.6 to 2.4 g with 95% margins of error of ∼0.4–0.8 g (Table 3).

### Monte Carlo EGL simulation

**FIGURE 2:**
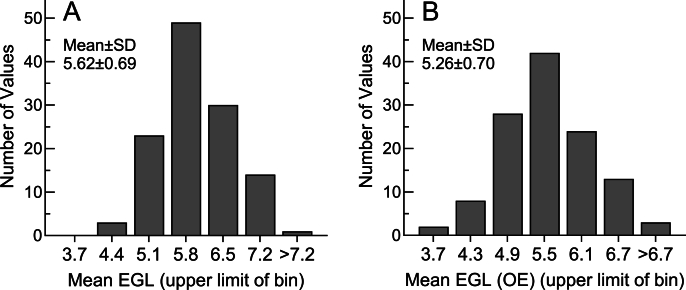
Distribution of mean EGL values from the Monte Carlo simulation. (A) Distribution of 120 mean EGL values (each consisting of 25 individual EGL values) representing the simulated mean EGL for WB5.2. (B) Distribution of 120 mean EGL values (each consisting of 22–25 individual EGL values) representing the simulated mean EGL for WB5.2 after excluding outliers. EGL, equivalent glycemic load.

The 3000 values generated to represent the iAUC elicited by WB20.8 were normally distributed (*P* = 0.997) with a mean ± SD of 99.8 ± 20.9 and the 3000 values generated to represent the iAUC elicited by WB5.2 were normally distributed (*P* = 0.26) with a mean of 25.7 ± 14.6 (Supplemental Figure 3A and 3B, respectively). The 3000 EGL values generated for WB5.2 had a mean ± SD of 5.62 ± 3.53 g and were not normally distributed (*P* < 0.001) (Supplemental Figure 4A). After excluding 131 outliers (123 above the mean and 8 below the mean) the remaining 2869 values were normally distributed (*P* = 0.32) and had a mean ± SD of 5.26 ± 3.01 g (Supplemental Figure 4B). The means of the 120 sets of 25 simulated EGL values were normally distributed both before and after excluding outlying values with means ± SDs of 5.62 ± 0.69 and 5.26 ± 0.70, respectively (Figure 2A, B, respectively).

The ratio of outliers to nonoutliers in the simulation, 131/2896, did not differ significantly from those measured in the 25 subjects, 11/189 (*P* = 0.45 by chi-square test). In the simulation, the distribution of the number of groups of 25 with 0, 1, 2, or 3 excluded outliers, respectively (22, 67, 29, and 2) did not differ significantly from those for the 8 foods (0, 5, 2, and 0, *P* = 0.63). Each subject tested 8 foods; 1 subject had 5 outliers, 2 subjects had 2 outliers, 2 subjects had 1 outlier, with the other 20 having no outliers.

### Plasma glucose and serum-insulin increments in 10 participants

The incremental glycemic and insulinemic responses elicited by the 10 test-meals in the 10 subjects in whom both glucose and insulin were measured are shown in Figure 1B, C (the plasma glucose and serum-insulin concentrations are shown in Supplemental Figure 2B, C). The mean glucose and insulin iAUCs for WB20.8#1 and WB20.8#2 did not differ from each other and were significantly greater than those for all the other test-meals. Mean glucose iAUC after WB5.2 was significantly greater than those for all 7 test-products, but there were no significant differences among the test-products (Table 4). Mean insulin iAUC after WB20.8#1 and WB20.8#2 did not differ from each other and were significantly greater than those for all the other test-meals. Mean insulin iAUC after CinnCer was significantly greater than those after PBDCB, ABBB, ADCB, and WB5.2 (Table 4).

**TABLE 4:** Incremental areas under the glucose and insulin curves in 10 participants

Test-meal	Abbreviation	Glucose (mmol × min/L)	Insulin (pmol × h/L)
White bread #1 (20.8 g avCHO)	WB20.8#1	100.4 ± 10.2^a^	228 ± 32^a^
White bread #2 (20.8 g avCHO)	WB20.8#2	121.5 ± 12.1^a^	275 ± 40^a^
White bread (5.2 g avCHO)	WB5.2	21.6 ± 2.9^b^	50 ± 14^c^
Sriracha crisp	SirCr	8.1 ± 2.7^c^	82 ± 19^bc^
Cheddar crisp	ChdCr	10.1 ± 3.3^c^	96 ± 16^bc^
Almond blueberry butter bar	ABBB	7.3 ± 2.4^c^	59 ± 10^c^
Peanut butter dark chocolate bar	PBDCB	6.6 ± 2.5^c^	64 ± 15^c^
Almond dark chocolate bar	ADCB	2.7 ± 1.5^c^	58 ± 12^c^
Peanut butter cereal	PBCer	8.0 ± 2.7^c^	82 ± 12^bc^
Cinnamon almond cereal	CinnCer	4.8 ± 1.9^c^	132 ± 26^b^

Results are mean ± SEM in 10 subjects.

^abc^Means not sharing the same letter superscript differ significantly by 2-tailed Tukey’s test (*P* < 0.05).

### Nutritional determinants of glucose and insulin iAUC elicited by the test-products

**FIGURE 3:**
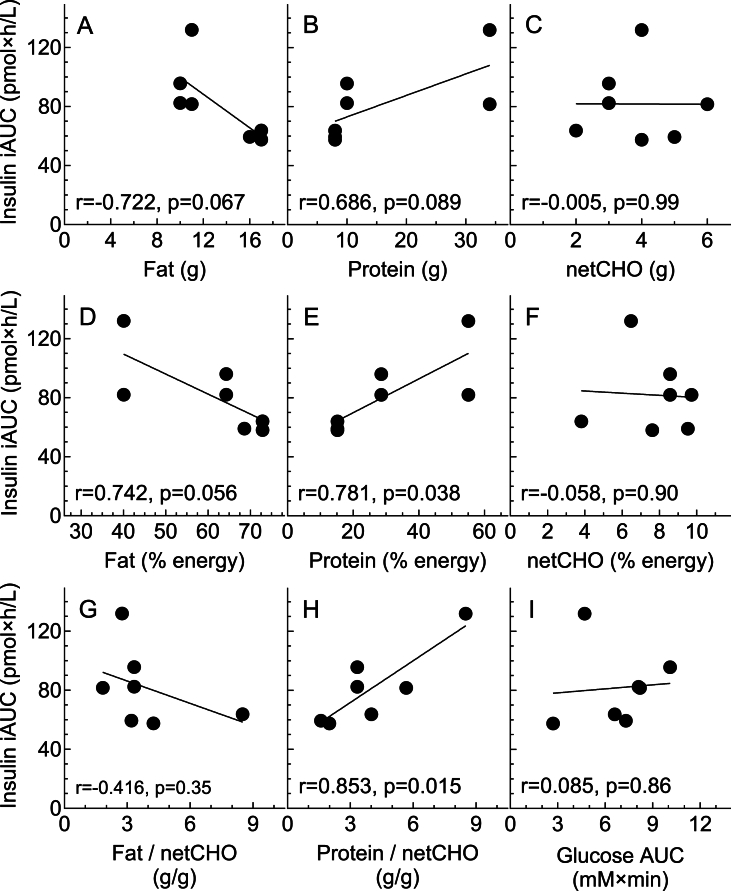
Nutritional determinants of insulin iAUC. Correlations between the mean incremental area under the curve for insulin (insulin iAUC) elicited by 1 serving of the 7 test-products in 10 healthy participants and the amounts of nutrients they contain per serving: (A) grams of fat; (B) grams of protein; (C) grams of netCHO (total carbohydrate minus dietary fiber minus allulose); (D) % energy from fat; (E) % energy from protein; (F) % energy from netCHO; (G) grams fat per gram of netCHO; (H) grams protein per gram of netCHO; and (G) glucose iAUC. *r* = correlation coefficient; *P* = significance of correlation; black lines are the regression lines.

The avCHO content of the test-products was taken to be total carbohydrate minus dietary fiber, and their “net-carbohydrate” (netCHO) content to be avCHO minus allulose (allulose was the only nonfiber, unavailable carbohydrate ingredient in any of the products; Table 1). Among the 7 test-products, in the 10 subjects in whom both glucose and insulin were measured, there was no significant correlation between glucose or insulin iAUC, respectively, and their total carbohydrate (*r* = −0.54, *P* = 0.22 and *r* = 0.07, *P* = 0.88) or avCHO contents (*r* = −0.41, *P* = 0.37 and *r* = 0.32, *P* = 0.48) (not shown). In addition, there was no significant correlation between mean glucose iAUC elicited by the 7 test-products and their content of energy (*r* = −0.55, *P* = 0.20, not shown), fat (*r* = −0.57, *P* = 0.18), protein (*r* = −0.07, *P =* 0.88), netCHO (*r* = −0.07, *P =* 0.88), % of energy from fat (*r* = −0.08, *P =* 0.86), protein (*r* = 0.12, *P =* 0.80) or netCHO (*r* = 0.38, *P =* 0.40), grams fat per gram netCHO (*r* = −0.17, *P =* 0.72), or grams protein per gram netCHO (*r* = −0.10, *P =* 0.83) (Supplemental Figure 5). There was also no significant correlation between mean glucose iAUC and mean insulin iAUC (*r* = 0.09, *P =* 0.86). By contrast, although there was no significant correlation between mean insulin iAUC and energy (*r* = 0.12, *P =* 0.80, not shown) or netCHO (Figure 3C, F), insulin iAUC tended to be positively related to protein grams per serving and significantly related to % energy from protein and protein per gram netCHO (Figure 3B, E, H). Insulin iAUC tended to decrease with increasing fat and % energy from fat but there was no significant correlation with fat per gram netCHO (Figure 3A, D, G).

## Discussion

The EGL of WB5.2 equals the amount of avCHO it contains, i.e. 5.2 g. We hypothesized that that measured EGL is accurate and precise to within ±1 g. The observed mean EGL of WB5.2, after excluding outliers, 4.0 g, was within 1.2 g of the actual value (5.2 g), and the 95% margin of error was 0.64 g. Because carbohydrate values on the NFL are rounded to the nearest gram, this result supports the hypothesis. It could be argued that outliers should not be excluded; however, the results without excluding outliers also support the hypothesis, because the mean EGL, 4.3 g, was within 0.9 g of the actual value with a 95% margin of error of 0.89 g.

Error in the measurement of avCHO could affect the accuracy of the EGL value obtained for WB5.2. On the basis of the SD of the 4 proximate analyses of WB we performed (Table 1), the 95% CI of the avCHO contents of WB20.8 and WB5.2, respectively, are 19.9, 21.7 g and 5.0, 5.4 g. However, it is unlikely that such variation could account for a mean EGL of 4.0 g because <0.1% of the EGLs obtained would be outside the range of 4.8–5.6 g.

Day-to-day variation of glycemic responses within individuals is substantial and could affect the accuracy of EGL values. The Monte Carlo simulation demonstrated that the distribution of the ratios of independently variable normally distributed values (Supplemental Figure 3) is skewed to the right (Supplemental Figure 4A) and falsely increases the mean and SD, as previously described [17,18,19]. Excluding outliers normalized the distribution (Supplemental Figure 4B) and reduced the mean (Figure 2) suggesting that excluding outliers provides a more accurate estimate of EGL. The validity of the model is suggested by the fact that the proportion and distribution of outliers excluded in the simulation did not differ significantly from those in the measured values. EGL values >1.96 SD above the mean may be due to an unusually high value in the numerator (the food iAUC) or an unusually low value in the denominator (the WB20.8 iAUC) or both. If the WB20.8 iAUC is unusually low, it would affect the EGL for all the foods tested, as is likely the case for the subject in whom there were 5 of 8 outlying values. If the food iAUC is unusually high, it would only affect the EGL for that food, and such is likely the case for the subjects with 1 or 2 outliers.

The EGL value for WB5.2 we obtained, 4.0 g, is <97.5% of the 120 simulated EGL values. Nevertheless, it is within ∼1 g of the true mean of 5.2 g. The glycemic impact of 1 g avCHO from bread, ∼5% of that elicited by WB20.8, is insignificant compared with the glycemic impact of within-individual variation, the SD of which was ∼28% of that elicited by WB20.8.

EGL is analogous to glycemic load (GL) except, where GL is the grams of glucose, EGL is the grams of avCHO from WB that elicits the same glucose iAUC as a serving of the food. GL is defined as GI × g/100 (GI = glycemic index and g = grams avCHO per serving of food); however, it is not possible to measure the GI of low-carbohydrate foods because, to do so, subjects must consume portions of food containing ≥25 g avCHO within 10–15 min [19]. The 25 g avCHO portions of low-carbohydrate foods are larger than most subjects can comfortably consume at 1 time; for the foods tested here such portions would contain 1070–2730 kcal. WB is used as the reference for EGL because the GI of WB is similar to that of most starchy foods consumed in North America and, hence, is more physiologically relevant than glucose.

A potential drawback of using WB is that it may be less reproducible than glucose due to differences in flour, baking, removal of crusts, and storage. However, the results of an interlaboratory GI study involving centers in Canada, South Africa, Sweden, Australia, Italy, New Zealand, and Trinidad and Tobago [20] suggest that such differences may not be large. The participating laboratories measured the GI of samples of instant potato, rice, spaghetti, and barley sent to them from Toronto along with instructions about portion preparation. Because WB could not be sent from Toronto, centers tested their local WB. The SD of the mean GI of the 7 laboratories for bread, 6.4, was 30%–50% less than that for the 4 standardized foods (9.6–12.1) [20].

The glucose iAUC elicited by conventional high-carbohydrate foods (e.g. sugars, grains, tubers, and legumes) is determined by the source and amount of avCHO consumed [3,4]. Here, the test-products contained 3–12 g avCHO (NFL values for carbohydrate minus fiber) but had EGL values ranging from only 0.6 to 2.4; i.e. 20%–90% less than might be expected from the NFL. This is partly because several of the test-products were sweetened with allulose (also known as D-psicose), an epimer of fructose [21,22]. Although well absorbed, allulose is not metabolized and is excreted in the urine [21]. Allulose is an example of a partly or totally unavailable carbohydrate that, on the NFL, is not included in the value for fiber but is included in carbohydrate. Although listed in the ingredient list, the amount in a serving of food is often not indicated on the NFL. In the foods tested here, the amount of truly avCHO can be estimated as ”carbohydrate” minus “fiber” minus allulose, i.e. 3–6 g (Table 1); such values represent what is often termed netCHOs [23].

However, the EGL values of the 7 test-products were also 20%–85% less than their content of netCHO. This is likely because the test-products were high in fat and protein, which, together, accounted for 85% or more of their energy content. Adding fat and/or protein to carbohydrate reduces the glycemic response by delaying gastric emptying and increasing postprandial insulin [[12], [13], [14]]. Adding fat to avCHO reduces the glucose iAUC, in a log-linear fashion with each gram of added fat per gram avCHO (g/g) reduces glucose iAUC by 46% (95% CI: 35, 65) [24]; similarly, each g/g added protein reduces glucose iAUC by 50% (95% CI: 42, 57) and increases insulin by 51% (95% CI: 29, 77) [25]. However, it is not possible to calculate the g/g effects of fat and protein on postprandial glucose and insulin responses in present study because the test-products contained both protein and fat and the glycemic impact of the avCHOs in the test-products in the absence of protein and fat is unknown.

The lack of a significant correlation between glucose iAUC and the netCHO contents of the 7 test-products (Supplemental Figure 5C, F) should not be interpreted to mean that netCHO is not a determinant of glycemic response. The lack of correlation here is likely due to a combination of factors including the small number of products tested, the small amounts of netCHO they contained, the small range of their glycemic responses (2.7–10.1 mmol × min/L in the face of 95% margins of error of 3.1–6.7 mmol × min/L), and the confounding effects of the large differences in their protein (8–34 g) and fat (10–17 g) contents.

As expected, the mean glucose and insulin iAUC after WB5.2 were both ∼20% of the means for WB20.8. However, as the dose of avCHO increases beyond 20 g, the dose-response curve for glucose iAUC begins to flatten off, whereas that for insulin iAUC continues to increase linearly [12,13]. Consistent with the effect of adding protein to avCHO [25], the insulin responses of the test-products were positively related to their protein content. The health implications of differences in such small insulin responses are not clear. Over 30 y ago, elegant studies from DeFronzo’s group showed that a 72 pmol/L increase in serum insulin sustained for 3 d induced insulin resistance and reduced whole body glucose disposal and the ability of hyperglycemia to induce insulin secretion [26]. Several of the test-products increased mean serum insulin by >72 pmol/L (Figure 1C), and the iAUC after CinnCer was 72 pmol × min/L higher than that after WB5.2 (Table 3). On the other hand, the risk of type 2 diabetes is not affected by protein intake [27], with the possible exception of red meat [28].

Overestimation of the amount of avCHO in a low-carb food may be a concern for people with type 1 diabetes (T1D) who might overestimate the amount insulin required, possibly leading to hypoglycemia. However, the amount of avCHO overestimated is small and may not be important; this warrants further study. Of more concern, perhaps, is that fact that low-carb foods are often high in protein which, in T1D, increases postprandial glucose when added to carbohydrate and/or fat [29]. For example, in T1D, adding 12.5, 25, 50, or 75 g whey protein to 30 g glucose consumed 4,h after a standardized evening meal elicited a delayed rise in postprandial glucose that, with the highest doses, continued to increase over the 5-h study period [30].

Subjects could choose to have with their test-meals, a drink of water, tea or coffee with or without 30 mL of 2% milk, and a noncaloric sweetener. This could be criticized because the different drinks may have different effects on postprandial responses. However, because the same drink was taken with every test-meal taken by each subject, their relative effects on postprandial responses were controlled for. Indeed, we found that, compared with drinking water with all test-meals, drinking coffee or tea had no significant effect on the mean GI, but reduced the SD of the GIs by ∼30% [31].

We conclude that the results support the hypothesis that the EGL measure is accurate and precise to within ∼±1 g of netCHO. The EGLs of the 7 test-products were 20%–90% less than might be expected from their food-labels. The test-products elicited small insulin responses that were positively related to their protein content.

## Author contributions

The authors’ responsibilities were as follows – TMSW: developed the details of the research plan, provided study oversight at INQUIS, analyzed the data, performed the statistical analysis, wrote the paper, and had primary responsibility for its final content; KM: conceived of the project and assisted in developing the research plan, provided essential materials (the test-meals and their nutritional content), and provided project oversight at General Mills until he left the company; TB: took over the provision of project oversight at General Mills; KM, TB: provided valuable comments on the first draft of the paper; and all authors: have read and approved the final manuscript.

## Data availability

Deidentified participant data pertaining to glucose and insulin measurements will be available after publication upon reasonable request and will be available until 3 y after the publication date.

## Funding

Funding for the study was provided by General Mills, Inc. KM and TB assisted in the design of the project, provided the test foods, reviewed and approved the protocol, and reviewed and commented on manuscript drafts. However, TMSW determined the final wording in the manuscript. Before starting the study, the sponsor indicated its wish to have the results published and had no restrictions regarding publication.

## Conflict of interest

TMSW reports a relationship with INQUIS Clinical Research Ltd that includes employment. KM reports a relationship with General Mills, Inc that includes employment. TB reports a relationship with General Mills, Inc that includes employment. TMSW received reimbursement of travel and accommodation expenses from General Mills to attend the American Society of Nutrition annual meeting in 2024 at which he was invited by General Mills to deliver a lecture at an accredited General Mills sponsored symposium. The honorarium for this talk was paid to INQUIS Clinical Research and did not affect TMSW’s salary.
