# Supplementary material for: Equivalent Glycemic Load and Insulinemic Responses Elicited by Low-Carbohydrate Foods: A Randomized Trial in Healthy Adults
Source: Curr Dev Nutr. 2025 Oct 30;9(12):107594. doi: 10.1016/j.cdnut.2025.107594 (PMC12719656; doi:10.1016/j.cdnut.2025.107594)
Supplement: Multimedia component 1 [file mmc1.docx]

# Contents

[Contents 1](#_Toc208917506)

[1. Screening 2](#_Toc208917507)

[1.1 Inclusion/exclusion/withdrawal criteria 2](#_Toc208917508)

[1.2 Tool for determining Sex and Gender 3](#_Toc208917509)

[1.3 Tool for determining Ethnicity 3](#_Toc208917510)

[2. Results 4](#_Toc208917511)

[2.1 Medications used by participants. 4](#_Toc208917512)

[2.2 Adverse events. 4](#_Toc208917513)

[2.3 Protocol deviations. 4](#_Toc208917514)

[2.4 Plasma Glucose Analysis 4](#_Toc208917515)

[2.5 Serum Insulin Analysis 4](#_Toc208917516)

[Supplementary Figures 5](#_Toc208917517)

[Supplementary Figure 1: Study Diagram 5](#_Toc208917518)

[Supplementary Figure 2: Plasma glucose and insulin concentrations 6](#_Toc208917519)

[Supplementary Figure 3: Distribution of iAUC values from the Monte Carlo simulation 7](#_Toc208917520)

[Supplementary Figure 4: Distribution of individual EGL values from the Monte Carlo simulation 7](#_Toc208917521)

[Supplementary Figure 5: Nutritional determinants of glucose iAUC 8](#_Toc208917522)

# 1. Screening

## 1.1 Inclusion/exclusion/withdrawal criteria

**Inclusion criteria:**

- Adults aged 18-75, inclusive.
- Willing to abstain from unusual strenuous exercise and consuming alcoholic drinks for 24 hours before study days.
- Willing to refrain from smoking tobacco or marijuana for 12h before and during study visits.
- Understand the study procedures and willing to provide informed consent to participate in the study and authorization to release relevant protected health information to the study investigator.
- Subjects must be eligible to receive income in Canada and be covered by a health insurance plan such as OHIP.

**Exclusion and withdrawal criteria:**

- Failure to meet any one of the inclusion criteria at screening.
- Pregnant or lactating individuals.
- Personal history of diabetes.
- Major trauma or surgical event within 3 months of screening.
- The presence of any laboratory result, health condition, illness or drug use that increases risk to the subject or to others or may affect the results, as judged by the Qualified Investigator.
- Unwillingness or inability to comply with experimental procedures and to follow INQUIS safety guidelines.
- Known intolerance, sensitivity, or allergy to any ingredients in the study test meals. All allergens should be assumed to be a result of manufacturing practices.
- Subject is currently participating or recently (within 30 days of screening) participated in a clinical trial involving long-term exposure (greater than 24 hours) to an investigational drug, nutritional supplement, or lifestyle modification.

**Withdrawal Criteria:**

- Withdrawal of consent for any reason
- The development, during the study, of an exclusion criterion, injury, illness or initiation of use of a medication which, in the opinion of the Qualified Investigator, makes the subject's continued participation dangerous to the subject or to others, or which may affect the results.
- Failure to follow INQUIS safety guidelines.
- Repeated failure to attend scheduled visits.
- Failure to follow the protocol.
- Lost to follow-up.
- Investigator elects to remove participant from the study for any reason.

## 1.2 Tool for determining Sex and Gender

| C1. Assigned Sex at Birth | | C2. Gender Identity: | |
| --- | --- | --- | --- |
|  | 1. Male |  | 1. Male |
|  | 2. Female |  | 2. Female |
|  | 3. Intersex |  | 3. Intersex |
|  |  |  | 4. Transgender Male to Female |
|  | |  | 5. Transgender Female to Male |
|  |  |  | 6. Prefer not to answer |
|  |  |  | 7. Do not know |
|  |  |  | 8. Other (Please specify): |
|  |  |  | ____________________________________ |

## 1.3 Tool for determining Ethnicity

D1. Ethnicity (please select only one):

|  | 1. | Asian - East (e.g., Chinese, Japanese, Korean) |
| --- | --- | --- |
|  | 2. | Asian - South (e.g., Indian, Pakistani, Sri Lankan) |
|  | 3. | Asian - South East (e.g., Malaysian, Filipino, Vietnamese) |
|  | 4. | Black - African (e.g., Ghanaian, Kenyan, Somali) |
|  | 5. | Black - North American (e.g., Canadian, American) |
|  | 6. | Black - Caribbean (e.g., Barbadian, Jamaican) |
|  | 7. | First Nations - Non-status |
|  | 8. | First Nations - Status |
|  | 9. | Indian - Caribbean (e.g., Guyanese with origins in India) |
|  | 10. | Indigenous/Aboriginal not included elsewhere |
|  | 11. | Inuit |
|  | 12. | Latin American (e.g., Argentinean, Chilean, Salvadorian) |
|  | 13. | Métis |
|  | 14. | Middle Eastern (e.g., Egyptian, Iranian, Lebanese) |
|  | 15. | White - European (e.g., English, Italian, Portuguese, Russian) |
|  | 16. | White - North American (e.g., Canadian, American) |
|  | 17. | Mixed heritage (Please specify): ____________________________________________ |
|  | 18. | Other (Please specify): ____________________________________________________ |

# 2. Results

## 2.1 Medications used by participants.

Fifteen (15) participants took prescription (Rx), over the counter (OTC) drugs, natural health products (NHP) and/or supplements; they comprised 7 of 10 participants in whom insulin was measured (IM) and 8 of 15 participants in whom only glucose was measured (OG, p=0.40).

IM participants: ID#4 took 10mg loratadine as needed (PRN) for allergies; ID#8 took 2 types of protein (7.5 and 54g) shakes 3 times weekly; ID#9 applied 1% hydrocortisone cream PRN for eczema; ID#13 took 1000IU vitamin D daily; ID#15 took a high protein shake (62g) PRN for general health and 10mg loratadine PRN for allergies; ID#18 took 5g creatine and 1.25mg finasteride daily, 10/240mg loratadine/pseudo-ephedrine PRN for allergies, and 500mg acetaminophen PRN; and ID#20 took 120μg thyroxine, 40mg pantoprazole, 50mg atenolol and 20mg rosuvastatin daily.

OG participants: ID#6 took 25mg diphenhydramine PRN for allergies; ID#10 took 500mg acetaminophen and 60/120mg Fexofenadine/pseudoephedrine for one day, 2 days before a study visit; ID#11 applied 5% permethrin cream PRN for eczema and took acyclovir for 6 days for a cold sore; ID#16 took 75mg venlafaxine and 500mg valacyclovir daily, and 220mg naproxen PRN; ID#17 took a multivitamin, 1000IU vitamin D and 1000mg vitamin C and zinc daily and 200mg ibuprofen PRN; ID#19 took 10mg loratadine PRN for allergies; ID#23 took 5g creatine PRN for general health and ID#26 had seasonal allergy injections once per month.

## **2.2 Adverse events**.

ID#4 had a cut knee which healed without treatment. ID #10 had a sore throat and congestion, treated with OTC medications, that resolved in a few days. ID#11 had a cold sore that was treated with a topical antiviral and resolved in a few days. ID#24 experienced congestion and fatigue and ID#25 had a cold; both resolved without treatment in 3-5 days and the planned study visits were postponed until the participants had recovered.

## 2.3 Protocol deviations.

There were n=21 minor protocol deviations (PD) in n=7 participants For n=8 PDs the participant fasted for 15-16.5h instead of 10-14h; for n=3 the participant ate the test meal over 8-11min instead of 12-15min; 1 glucose and insulin sample (n=2 PDs) was taken at 75min instead of 90min and a sample for glucose and insulin (n=2 PDs) was taken at 75min instead of 60min (the iAUC calculations were adjusted to account for these time differences) and 1 glucose sample took 6.5min to collect and another was left at room temperature for 15min before spinning; one participant, who chose to have water with the test meals, was accidentally given water containing dextrose with the WB5 test meal – after this was discovered the WB5 the test meal was repeated resulting in the repeated WB5 meal not following the randomization sequence (n=3 PDs); 1 participant consumed an extra 100ml water due to the spicy test meal.

## 2.4 Plasma Glucose Analysis

None of the 2250 glucose values was missing. The mean±SD of the 250 pairs of duplicate analyses of the 0 min sample was 5.42±0.034 mmol/L for a CV of 0.64% which represents analytical variation. The mean±SD of 250 pairs of -5- and 0-min samples was 5.44±0.090 mmol/L for a CV of 1.7% which includes both minute-to-minute and analytic variation.

## 2.5 Serum Insulin Analysis

None of the 800 insulin values was missing. The mean±SD of the 100 pairs of -5- and 0-min samples were 73.8±20.6 pmol/L, a value that includes both analytic variation and minute-to-minute variation.

# Supplementary Figures

## Supplementary Figure 1: Study Diagram


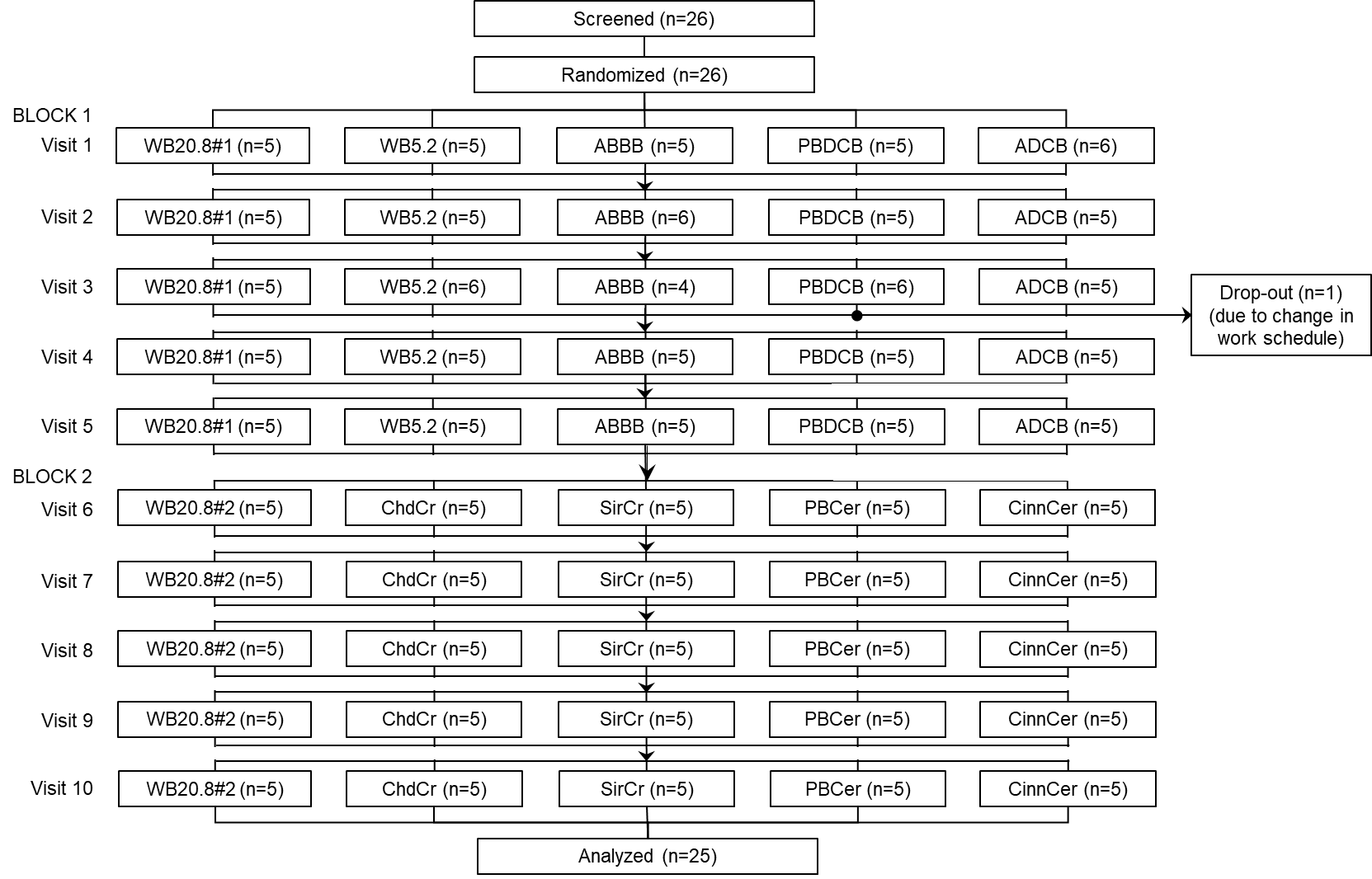


WB20=white bread containing 20.8g available carbohydrate; WB5=white bread containing 5.2g available carbohydrate; ABBB=Almond blueberry butter bar; PBDCB=Peanut butter dark chocolate bar; ADCB=Almond dark chocolate bar; ChdCr=Cheddar crisp; SirCr=Siracha crisp; PBCer=Peanut butter cereal; CinnCer=Cinnamon almond cereal.

## Supplementary Figure 2: Plasma glucose and insulin concentrations

Values are means±SEM (error bars not shown if they overlap or are smaller than the symbol). Panel A: plasma glucose concentrations in n=25 subjects. Panel B: plasma glucose concentrations in n=10 subjects. Panel C: serum insulin concentrations in the same n=10 subjects as shown in panel B. WB20.8#1 and WB20.8#2, 20.8g avCHO portions of white bread tested in the first and second phases, respectively; WB5, 5g avCHO portion of white bread; ABBB, almond blueberry butter bar; ADCB, almond dark chocolate bar; PBDCB, peanut butter dark chocolate bar; ChdCr, cheddar crisp; SirCr, siracha crisp; CinnCer, cinnamon cereal; PBCer, peanut butter cereal.

## Supplementary Figure 3: Distribution of iAUC values from the Monte Carlo simulation

iAUC = incremental area under the curve ignoring area below fasting glucose.

Panel A: Distribution of n=3000 normally distributed random numbers representing the within-individual variation of the incremental area under the glucose curve (iAUC) elicited by WB20.8.

Panel B: Distribution of n=3000 normally distributed random numbers representing the within-individual variation of the incremental area under the glucose curve (iAUC) elicited by WB5.2.

## Supplementary Figure 4: Distribution of individual EGL values from the Monte Carlo simulation

EGL = equivalent glycemic load.

Panel A: Distribution of n=3000 individual values representing the simulated EGL for WB5.2.

Panel B: Distribution of n=2877 individual values, after excluding n=123 outliers >1.96×SD from the mean, representing the simulated EGL for WB5.2 after excluding outliers.

## Supplementary Figure 5: Nutritional determinants of glucose iAUC

Correlations between the mean incremental area under the curve for glucose (glucose iAUC) elicited by 1 serving of the 7 test-products in n=10 healthy participants and the amounts of nutrients they contain per serving: Panels: **A**, fat; **B**, protein; **C**, netCHO (total carbohydrate minus dietary fiber minus allulose); **D**, % energy from fat; **E**, % energy from protein; **F,** % energy from netCHO; **G**, grams fat per gram of netCHO; **H**, grams protein per gram of netCHO; and **G** insulin iAUC. r = correlation coefficient; p=significance of correlation; black lines are the regression lines.
